# Toward identifying reproducible brain signatures of obsessive-compulsive profiles: rationale and methods for a new global initiative

**DOI:** 10.1186/s12888-020-2439-2

**Published:** 2020-02-14

**Authors:** Helen Blair Simpson, Odile A. van den Heuvel, Euripedes C. Miguel, Y. C. Janardhan Reddy, Dan J. Stein, Roberto Lewis-Fernández, Roseli Gedanke Shavitt, Christine Lochner, Petra J. W. Pouwels, Janardhanan C. Narayanawamy, Ganesan Venkatasubramanian, Dianne M. Hezel, Chris Vriend, Marcelo C. Batistuzzo, Marcelo Q. Hoexter, Niels T. de Joode, Daniel Lucas Costa, Maria Alice de Mathis, Karthik Sheshachala, Madhuri Narayan, Anton J. L. M. van Balkom, Neeltje M. Batelaan, Shivakumar Venkataram, Anish Cherian, Clara Marincowitz, Nienke Pannekoek, Yael R. Stovezky, Karen Mare, Feng Liu, Maria Concepcion Garcia Otaduy, Bruno Pastorello, Rashmi Rao, Martha Katechis, Page Van Meter, Melanie Wall

**Affiliations:** 1 Columbia University Irving Medical Center, Columbia University, New York, NY 10032 USA; 2 The New York State Psychiatric Institute, New York, NY 10032 USA; 3 Department of Psychiatry, Amsterdam UMC, Vrije Universiteit Amsterdam, de Boelelaan 1117, Amsterdam, Netherlands; 4 Department of Anatomy and Neuroscience, Amsterdam UMC, Amsterdam Neuroscience, Vrije Universiteit Amsterdam, de Boelelaan 1117, Amsterdam, Netherlands; 5 Obsessive-Compulsive Spectrum Disorders Program, Institute & Department of Psychiatry, Hospital das Clinicas-HCFMUSP, University of Sao Paulo Medical School, Sao Paulo, Brazil; 6 National Institute of Developmental Psychiatry, Sao Paulo, Brazil; 7 National Institute of Mental Health & Neurosciences (NIMHANS), Bangalore, India; 8 SAMRC Unit on Risk & Resilience in Mental Disorders, Department of Psychiatry & Neuroscience Institute, University of Cape Town, Cape Town, South Africa; 9 SAMRC Unit on Risk & Resilience in Mental Disorders, Department of Psychiatry, Stellenbosch University, Stellenbosch, South Africa; 10 Department of Radiology and Nuclear Medicine, Amsterdam UMC, Vrije Universiteit Amsterdam, de Boelelaan 1117, Amsterdam, Netherlands; 11 Amsterdam UMC, Vrije Universiteit, Psychiatry, Amsterdam Public Health Research Institute, de Boelelaan 1117, Amsterdam, Netherlands; 12 GGZ inGeest, Specialised Mental Health Care, Amsterdam, The Netherlands; 13 Institute of Radiology, Hospital das Clinicas-HCFMUSP, University of Sao Paulo Medical School, Sao Paulo, Brazil

**Keywords:** Obsessive-compulsive disorder, Neuroimaging, fMRI, Unaffected siblings, Brain signatures, Neurocognitive, Global mental health

## Abstract

**Background:**

Obsessive-compulsive disorder (OCD) has a lifetime prevalence of 2–3% and is a leading cause of global disability. Brain circuit abnormalities in individuals with OCD have been identified, but important knowledge gaps remain. The goal of the new global initiative described in this paper is to identify robust and reproducible brain signatures of measurable behaviors and clinical symptoms that are common in individuals with OCD. A global approach was chosen to accelerate discovery, to increase rigor and transparency, and to ensure generalizability of results.

**Methods:**

We will study 250 medication-free adults with OCD, 100 unaffected adult siblings of individuals with OCD, and 250 healthy control subjects at five expert research sites across five countries (Brazil, India, Netherlands, South Africa, and the U.S.). All participants will receive clinical evaluation, neurocognitive assessment, and magnetic resonance imaging (MRI). The imaging will examine multiple brain circuits hypothesized to underlie OCD behaviors, focusing on morphometry (T1-weighted MRI), structural connectivity (Diffusion Tensor Imaging), and functional connectivity (resting-state fMRI). In addition to analyzing each imaging modality separately, we will also use multi-modal fusion with machine learning statistical methods in an attempt to derive imaging signatures that distinguish individuals with OCD from unaffected siblings and healthy controls (Aim #1). Then we will examine how these imaging signatures link to behavioral performance on neurocognitive tasks that probe these same circuits as well as to clinical profiles (Aim #2). Finally, we will explore how specific environmental features (childhood trauma, socioeconomic status, and religiosity) moderate these brain-behavior associations.

**Discussion:**

Using harmonized methods for data collection and analysis, we will conduct the largest neurocognitive and multimodal-imaging study in medication-free subjects with OCD to date. By recruiting a large, ethno-culturally diverse sample, we will test whether there are robust biosignatures of core OCD features that transcend countries and cultures. If so, future studies can use these brain signatures to reveal trans-diagnostic disease dimensions, chart when these signatures arise during development, and identify treatments that target these circuit abnormalities directly. The long-term goal of this research is to change not only how we conceptualize OCD but also how we diagnose and treat it.

## Background

Obsessive-compulsive disorder (OCD) has a lifetime prevalence of 2–3% [1, 2] and results in reduced quality of life [3, 4], lower educational attainment [5], suicidality [6, 7], and even premature death [8, 9]. A leading global cause of disability [10, 11], OCD contributes to significant economic burden [12] that is expected to increase over the next 20 years [13]. Even among individuals who receive evidence-based treatments [14], only some achieve remission [15, 16]. One contributor to poor outcomes might be variation in neurobiological mechanisms that underlie different symptom profiles; addressing this variation with targeted treatments should improve care.

To begin to address this public health challenge, we launched an international collaboration with two specific aims. Our first aim is to identify reproducible brain signatures that distinguish individuals with OCD from unaffected siblings and healthy control subjects. Our second aim is to link these brain signatures to neurocognitive and clinical profiles observed in individuals with OCD. This approach is consonant with the United States (U.S.) National Institute of Mental Health’s initiative on Research Domains Criteria (RDoC). The RDoC initiative seeks to develop a research classification system for psychopathology based on dimensions of neurobiology and observable behavior and to use these dimensions as targets for treatment development [17]. Our success could ultimately lead to the development of objective methods for diagnosing OCD and identifying new treatment targets for it, with relevance to diverse populations across the globe. Moreover, since some OCD symptom profiles overlap with those seen in anxiety disorders and other obsessive-compulsive-related disorders, the data generated by this study may pave the way for a transdiagnostic understanding of these brain-behavior associations and enable longitudinal studies that identify the point at which these brain signatures arise during development. This paper provides the rationale for our study design and describes our research methods.

### Rationale

#### Why study OCD?

The clinical hallmarks of OCD are obsessions and compulsions. Obsessions include repetitive unwanted thoughts, images, impulses, or urges that typically generate distress; compulsions are repetitive behaviors or mental acts that the individual feels driven to perform [18]. In addition, up to 60% of individuals with OCD experience sensory phenomena, which are defined as subjective experiences that precede compulsions, and can include physical sensations, just-right sensations, and feelings of incompleteness [19–21]. Across countries and cultures, obsessions and compulsions cluster around common themes known as symptom dimensions. These dimensions include: concerns about contamination and cleaning compulsions; fear of harm and checking compulsions; need for symmetry/exactness and repeating, ordering, and counting compulsions; and forbidden or taboo thoughts (e.g., aggressive, sexual, religious obsessions) and related compulsions [22–24]. Because the core behaviors that characterize OCD—obsessions and compulsions—are relatively stereotyped across countries and cultures, focusing on this disorder offers an excellent test of the ability to use objective methods to identify reproducible brain circuit abnormalities that are linked to this discrete psychopathology.

**Fig. 1:**
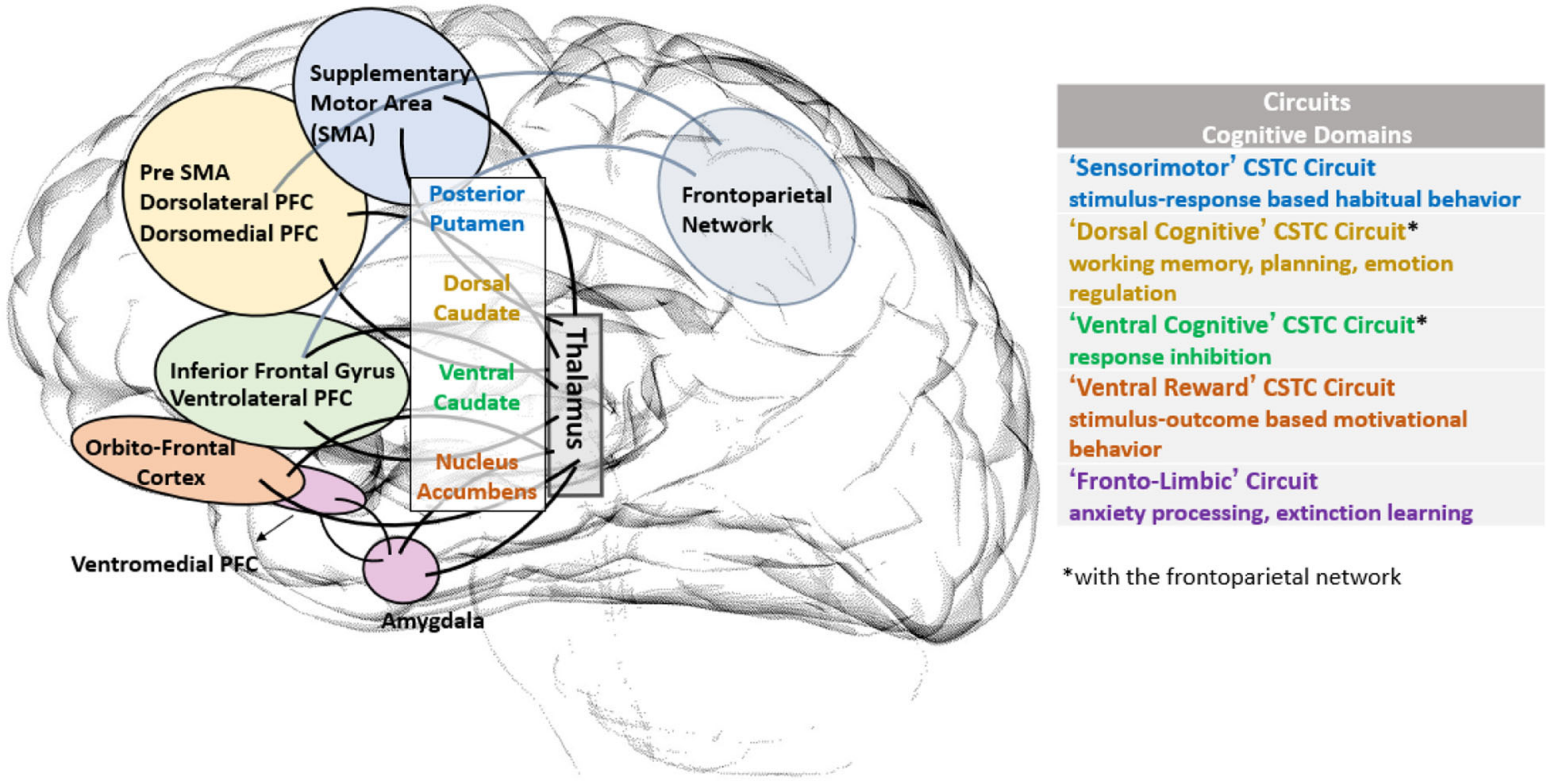
Multiple Brain Circuits Contribute to OCD

Another reason to focus on OCD is that the imaging literature has identified a relatively consistent pattern of brain circuit abnormalities related to the disorder. Specifically, dysregulation of cortico-striatal-thalamo-cortical (CSTC) circuits is thought to underlie OCD symptoms [25, 26]. Neuroimaging studies [27] have identified structural and functional abnormalities in multiple nodes of these CSTC circuits, including the frontal cortices, the striatum, and the thalamus [25, 28]. Abnormalities in limbic and fronto-parietal circuits have also been identified, and recent studies implicate the cerebellum [25, 26, 29–33]. These different circuits are depicted in Fig. 1, along with some of the key cognitive and behavioral processes that these circuits subserve [26].

However, prior imaging studies have important limitations that our study is designed to address. First, many have been single-site studies in small and historically Western, educated, industrialized, rich, and democratic (WEIRD) samples [27], and reproducibility of findings across sites has been variable. Meta- and mega-analyses (like those done by the OCD Brain Imaging Consortium (OBIC) and the initiative for Enhancing Neuroimaging and Genetics through Meta-analyses (ENIGMA) [34–37]) have been conducted, resulting in very large samples. These analyses pool existing data from multiple sites that use different inclusion criteria, clinical measures, and imaging methods. This variation not only introduces potential confounds, but also precludes linking brain findings to detailed cognitive or clinical profiles because of the lack of harmonization of such measures.

Second, most large-scale studies have been anatomical [33], examining brain volume only. Few large-scale studies have used diffusion weighted imaging (DWI) to assess structural connectivity [38–43] or resting state functional MRI (rs-fMRI) to assess functional connectivity [41, 44–54]. Moreover, existing DWI and rs-fMRI studies in OCD have used different acquisition and/or analytic methods, making lack of replication hard to interpret. In addition, many OCD subjects were on psychotropic medication at the time of imaging, despite the known effects on morphometry [37], DWI [55, 56], and rs-fMRI measures [47, 57].

Third, few studies have analyzed these imaging modalities in combination, despite the increasing recognition that multi-modal analysis of imaging data can help identify brain-behavior links [58]. Finally, although some single-site studies report significant correlations between brain circuit abnormalities (using DWI and rs-fMRI) and different neurocognitive [43] and clinical profiles [47, 59], the reproducibility of these findings needs rigorous testing across larger and more diverse populations using harmonized methods not only for brain imaging, but also for clinical phenotyping and neurocognitive testing [60].

**Fig. 2:**
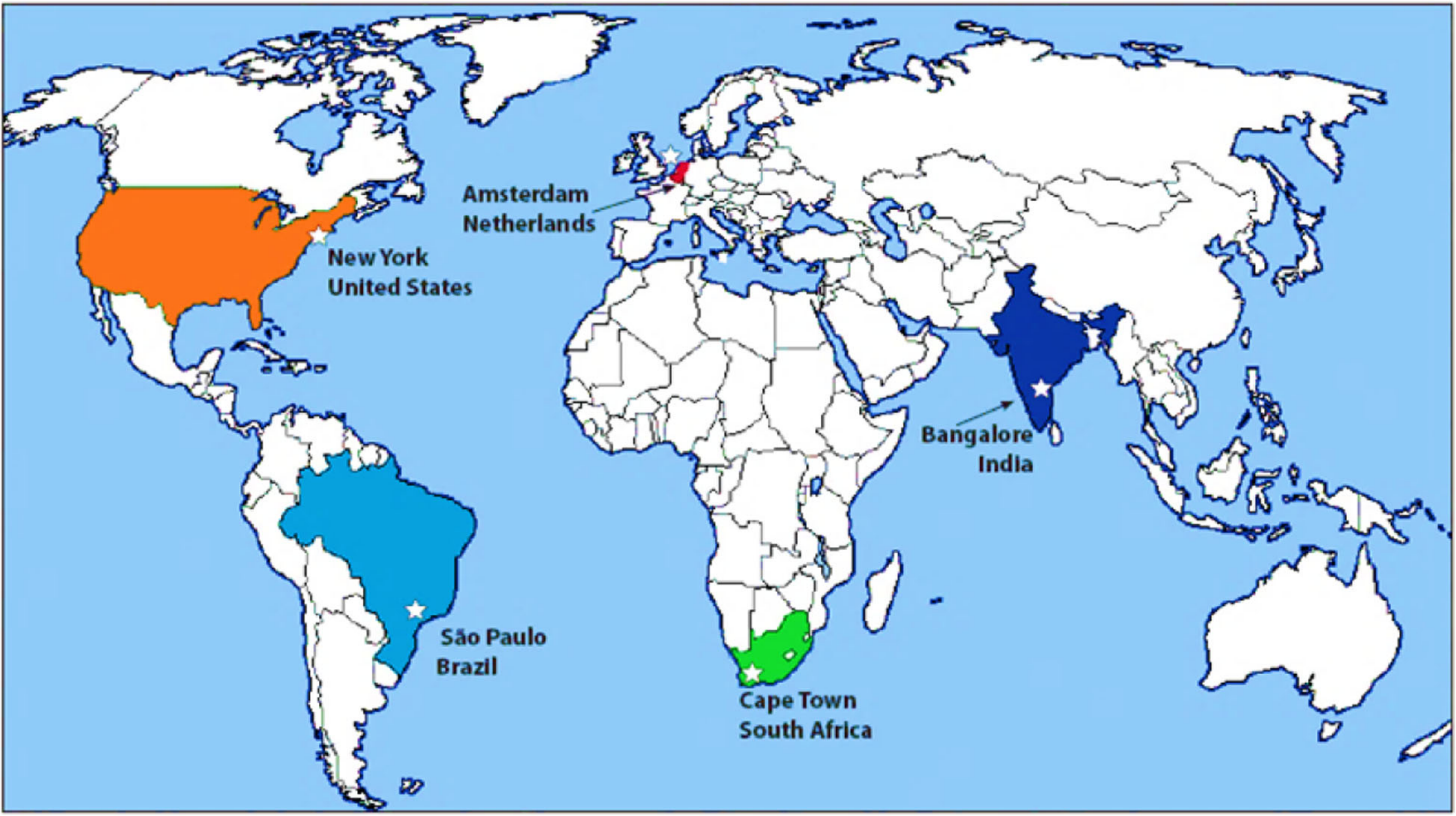
The Five Collaborating Sites

To address these limitations, we will recruit 250 medication-free individuals with OCD, 100 unaffected siblings of individuals with OCD, and 250 healthy control subjects (HCs) at five expert research sites that span five countries (Brazil, India, Netherlands, South Africa, U.S.; see Fig. 2). Using imaging methods chosen explicitly because of their potential adaptation for clinical use, we will examine multiple brain circuits thought to underlie OCD behaviors, focusing on morphometry (T1-weighted MRI), structural connectivity (DWI), and functional connectivity (rs-fMRI).

Our first aim is to identify imaging signatures that distinguish individuals with OCD from unaffected siblings and HCs; we will accomplish this by analyzing each modality with standardized protocols and by using multi-modal fusion with modern machine learning statistical methods. We hypothesize that individuals with OCD will show altered structure and function within specific frontal-striatal, frontal-limbic, and frontal-parietal circuits. Our second aim is to then link these imaging signatures both to behavioral performance on cognitive tasks that probe these same circuits and to discrete clinical profiles. We hypothesize that imaging signatures capturing different circuit abnormalities will correlate with behavioral performance on cognitive tasks that probe these same circuits as well as with discrete clinical profiles. Given the ethno-socio-cultural diversity of our sample, we will also explore how specific environmental features (childhood trauma, socioeconomic status, and religiosity) may moderate these brain-behavior links.

#### Why take a global approach?

Although OCD and its core features are observed across the globe, the specific presentation of OCD varies among individuals. Specifically, patients differ from one another both in the specific content of their OCD symptoms and in many other clinical features, including symptom severity, age of onset, course of illness, degree of insight, comorbidity, family history, and degree of functional impairment [61–64]. Some have reported that this variation in clinical presentation is reflected in different brain abnormalities [10, 27, 62, 65–68]. However, other differences in disease expression may be better explained by specific environmental and cultural factors. Therefore, recruiting a large, ethno-culturally diverse sample will enable us to test whether there are robust biosignatures of specific OCD clinical profiles that transcend countries and cultures and that could be used as targets for new treatments with relevance to populations across the globe.

Our sites were chosen for multiple reasons. First, all have expertise in OCD as well as the necessary clinical and imaging research infrastructure. Second, all can recruit an OCD sample that is similar in core OCD features and educational levels, yet diverse in socio-cultural features, enabling us to test how robust and replicable our imaging signatures are. Third, all can recruit medication-free OCD subjects in a timely and economical way, enabling us to collect this large sample within the funding boundaries of a single R01 from NIMH. Fourth, each is a center of excellence for research, training, and treatment of OCD in its respective country. Thus, our findings will have both local and global impact.

Finally, these sites had a successful track record of working together. For example, the Principal Investigators have worked together to revise the guidelines for the World Health Organization for OCD and Related Disorders [62] and have also collaborated in the ENIGMA-OCD consortium [69]. By joining forces for this study, we seek to shift the research model from local to global expertise, increase rigor and transparency, and accelerate discovery by developing a circuit-based approach to cognitive and clinical dimensions.

#### Why include siblings?

Given the high heritability of OCD [28], we will also include in our sample individuals who have a biological sibling with OCD but do not have OCD themselves (“unaffected” siblings). This approach has been used successfully in several prior imaging and neurocognitive studies in OCD [41, 54, 70–74]. Including unaffected siblings will support and strengthen our primary aims by allowing us to identify: (1) brain abnormalities that are present in OCD patients but not in their unaffected siblings or HC subjects (helping to define brain signatures that are most strongly linked to the disease state); (2) brain abnormalities that OCD patients and unaffected siblings share compared to matched HCs (helping to define brain signatures that may be linked to disease vulnerability); and (3) brain differences that are found in unaffected siblings but in neither HCs nor OCD patients (providing possible clues about resilience or compensatory brain mechanisms in unaffected siblings).

#### Why include environmental variables in a brain imaging study?

Some differences in OCD disease expression are hypothesized to be driven more by the local environment or contextual factors (e.g., relative severity of specific symptom dimensions, specific religious beliefs, degree of impairment/quality of life) than biology per se [75–78]. However, these factors have neither been examined in a large global sample, nor correlated with imaging and neurocognitive profiles. To begin to address this gap, we will use clinical assessments to explore disease expression across the sites. In addition, we will examine three specific environmental factors— childhood trauma, socioeconomic status (SES), and religiosity— to determine if they moderate the link between our neuroimaging signatures and clinical and cognitive profiles. We will focus on childhood trauma and SES because they have been identified as environmental risk factors for OCD [79–81]. Moreover, they have known effects on brain structure in healthy people [82–84], the potential to confound imaging data [83, 85–90], and have been positively associated with larger right orbitofrontal cortex volume in individuals with OCD [91]. Religiosity (defined as the salience of religious experience in a person’s life) has been associated with higher OCD symptoms, regardless of religion, and will thus also be explored within this multicultural study context. Finally, we will capitalize on this large and international sample to collect qualitative data with the Cultural Formulation Interview (CFI) [92] to explore the role of culture in the presentation and understanding of illness in those with OCD.

## Methods

### Study design

This project is an observational study that includes brain imaging and clinical and neurocognitive assessments, as described below.

### Setting(s)

This study is being conducted at five expert OCD research sites, including: the Obsessive-Compulsive Spectrum Disorders Program led by Drs. Euripedes Miguel and Roseli Shavitt at the Institute and Department of Psychiatry, Hospital das Clinicas-HCFMUSP, University of São Paulo Medical School in São Paulo, Brazil; the OCD program led by Dr. Janardhan Reddy at the National Institute of Mental Health and Neurosciences in Bangalore, India; the Neuropsychiatry section led by Dr. Odile van den Heuvel at the Department of Psychiatry/Anatomy & Neurosciences, Amsterdam University Medical Centers (location Free University (VU) Medical Center) in collaboration with the Anxiety and OCD Program of Mental Health Institute GGZ inGeest, Amsterdam, the Netherlands; the OCD program led by Drs. Dan Stein and Christine Lochner at the South African Medical Research Council Unit on Risk and Resilience in Mental Disorders in Cape Town, South Africa; and the Center for OCD and Related Disorders led by Dr. Helen Blair Simpson at the New York State Psychiatric Institute/Columbia University, New York, NY, USA. All sites have experience conducting OCD research as well as the necessary clinical research and imaging infrastructure (e.g., 3.0 T MRI machines).

This project uses a collaborative leadership model, with Dr. Simpson as the contact PI for NIMH. The executive committee is comprised of the principal investigators (PIs) at each site and their key personnel; each PI is responsible for the study at his or her site, and the committee meets by videoconference twice per month. Each site will recruit the same number of subjects and use harmonized methods for clinical assessment, neurocognitive testing, and imaging acquisition. The institutional review board or ethics board at each site (named above) has reviewed and approved the study procedures. All subjects will provide written informed consent prior to participation.

### Subjects

A total of 250 medication-free OCD patients, 100 unaffected siblings, and 250 healthy control subjects will be recruited across all five sites (50 OCD patients, 50 healthy control subjects, and 20 unaffected siblings per site). The OCD and healthy control samples will be matched on age, gender, and educational level (within and between sites). Inclusion and exclusion criteria are outlined in Table 1. OCD subjects must have OCD as their principal diagnosis with at least moderate severity. Unaffected siblings must have a first-degree sibling with OCD but not meet criteria for OCD themselves.

**Table 1:** Inclusion and Exclusion Criteria

	OCD (*n* = 250)	Unaffected Siblings (*n* = 100)	Healthy Controls (*n* = 250)
Inclusion Criteria	• 18–50 years old • Principal diagnosis of OCD • YBOCS ≥16	• 18–50 years old • Has sibling with OCD	• 18–50 years old
Exclusion Criteria	• Lifetime diagnosis of psychosis, bipolar disorder, anorexia, autism, or Tourette disorder • Current chronic tic disorder, substance-use disorder, binge-eating disorder, bulimia, or suicidality • Current use of psychotropic medications or CBT for OCD	• Current or lifetime psychiatric disorder other than MDD or anxiety disorders • Current use of psychotropic medications	• Current or lifetime psychiatric disorder other than MDD or anxiety disorders (if not in past year) • Current or past use of psychotropic medications • First-degree relative with OCD or tic disorder
	• Major medical or neurological diseases • IQ < 80 • Contraindications to MRI		

*OCD* Obsessive-compulsive disorder, *YBOCS* Yale-Brown Obsessive-Compulsive Severity Scale, *CBT* Cognitive-behavioral therapy, *IQ* Intelligence quotient, *MRI* Magnetic resonance imaging, *MDD* Major depressive disorder

### Measures

#### Screening for eligibility

To determine eligibility, a trained rater will conduct a clinical evaluation with the Structured Clinical Interview for DSM-5 (SCID) to confirm diagnosis, the Yale-Brown Obsessive-Compulsive Scale (Y-BOCS) [93, 94] to assess OCD severity, and an assessment of IQ. Screening will also include questions about treatment history, medical history, family psychiatric history, and tic disorder. Those eligible and interested will be enrolled after providing written informed consent.

Standardizing assessment of IQ presented a significant challenge given that no single IQ measure has been validated across all five countries and languages. Consequently, each site selected a measure of IQ that has been validated in the appropriate languages for its respective country and can yield a general IQ score as well as an estimate of performance and verbal domains (Brazil: Wechsler Abbreviated Scale of Intelligence First Edition (WASI-I) [95]; India: Binet Kamat Test [96]; Netherlands: selected subscales from the Wechsler Adult Intelligence Scale Fourth Edition (WAIS-IV) [97]; South Africa: Wechsler Abbreviated Scale of Intelligence Second Edition (WASI-II) [98]; USA: WASI-II). The IQ test will be administered by trained raters at each site.

#### Clinical evaluations

A standardized protocol will be used at all sites to clinically assess subjects in their respective language (i.e., Afrikaans, Dutch, English, Kannada, or Portuguese). This protocol will include the Common Data Elements required by NIMH as well as validated clinical measures that have been used around the globe and that tap different clinical profiles common in individuals with OCD. In addition, validated measures of trauma history, SES, and religiosity will also be used. These measures are shown in Table 2 and include semi-structured interviews performed by a trained rater and self-report questionnaires. We will also collect qualitative data using the Cultural Formulation Interview (CFI) [92] to explore the role of culture in the presentation and understanding of illness in those with OCD.

**Table 2:** Clinical Domains and Measures

Clinical Domains	Measures
Obsessive-Compulsive Profiles	
Total Severity	Yale-Brown Obsessive-Compulsive Scale (Y-BOCS) [93, 94]; Obsessive-Compulsive Inventory-R^a^ [99]
Dimension Severity	Dimensional Yale-Brown Obsessive-Compulsive Scale (DY-BOCS) [100]
Insight	Brown Assessment of Beliefs Scale (BABS) [101]
Sensory Phenomena	University of São Paulo Sensory Phenomena Scale (USP-SPS) [102]
Age of Onset	Structured Clinical Interview for DSM-5 (SCID) [103]; Center for OCD and Related Disorders Age of Onset and Course Form
Depression	Hamilton Depression Rating Scale (HAM-D) [104]
Anxiety	Hamilton Anxiety Rating Scale (HAM-A) [105]
Other Clinical Profiles	Autism Spectrum Quotient^a^ [106]; Center for OCD and Related Disorders Tic Questionnaire; Disgust Propensity and Sensitivity Scale^a^ [107]; Impulsive-Compulsive Behaviours Checklist^a^ [108]; Obsessive-Compulsive Personality Disorder Questionnaire
Functioning	World Health Organization Disability Assessment Schedule 2.0 (WHODAS) [109]
Environmental	
Socioeconomic Status	Work and Meaning Inventory (WAMI)^a^ [110];
Trauma	Childhood Trauma Questionnaire^a^ [111]
Religiosity	Religious Behaviors and Beliefs Questionnaire^a^ [112];

^a^Self-report measure; all other measures are administered by a trained clinician

#### Neurocognitive assessment

Trained experimenters at each site will assess subjects using a computerized neurocognitive protocol. Tasks were chosen that are: 1) valid (i.e., known to probe brain circuits and domains of cognitive dysfunction that are implicated in OCD (see Fig. 1); 2) generalizable (i.e., in the public domain and with minimal reliance on language); 3) reproducible (i.e., computerized and standardized); and 4) consonant with the NIMH’s RDoC matrix [113]. The brain circuits and domains of cognitive function that these tasks will probe are shown in Table 3. All tasks will be completed outside of the scanner.

**Table 3:** Cognitive Tasks and Domains

Brain Circuit	Cognitive Domain	Cognitive Task	Outcomes
Dorsal “cognitive” CSTC^a^	Working Memory	Visual Spatial N-Back [71]	Percent of correct trials overall and per condition
	Planning	Tower of London [114, 115]	Percent of correct trials overall and per task load
Ventral “cognitive” CSTC^a^	Response Inhibition	Stop-Signal [70, 116, 117]	Stop signal reaction time
Ventral “reward” CSTC	Reward Processing	Temporal Discounting [118–121]	Discount rate parameter on the intertemporal choice task [and risk aversion parameter on the risk aversion task]
Frontal-Limbic	Emotion Regulation / Executive Control	Emotional Stroop [115, 122]	Mean reaction time and Stroop effect
Sensorimotor CSTC	Motor Learning	Motor Sequencing [123–126]	Learning rate, speed and accuracy, variability in motor performance
Combination of CSTC	Reward learning / Decision Making	Two Stage Reinforcement Learning [127, 128]	Proportion of decisions to repeat a rewarded choice vs. an unrewarded choice following either a common or rare transition

^a^With the frontoparietal network

Although imaging studies in healthy subjects have shown that these tasks probe the brain circuits implicated in OCD, prior OCD studies have revealed mixed behavioral effects with these tasks, with only some finding behavioral deficits in individuals with OCD relative to healthy control subjects [129–131]. There are many potential reasons for these mixed results, including the fact that studies used different task versions and many tested OCD subjects who were taking medication and/or had different types of comorbid conditions. Moreover, not all prior studies measured and controlled for IQ [132]. Finally, if the behavioral deficits are subtle, they will be more difficult to detect consistently across samples, especially if samples are small. In the current study, we attempt to address these limitations by using computerized and standardized task versions, only testing subjects who are medication-free, measuring IQ, and recruiting a large sample.

#### MRI imaging

All subjects will undergo multi-modal MRI in a 3.0 Tesla whole-body scanner equipped with a 32-channel (or 48-channel at the U.S. site) phased-array head coil. Details about the MRI scanners and sequences are provided in Table 4. Sequences include: 1) high-resolution 3D T1 weighted structural imaging using MPRAGE according to the ADNI3 protocol with 1 mm isotropic resolution; 2) multi-shell diffusion weighted imaging (DWI), and 3) resting-state fMRI (10 min, eyes closed). In addition, we added a high-resolution 3D phase-sensitive inversion recovery (PSIR) sequence to optimize segmentation of deep grey matter structures. All image processing will be conducted by the Netherlands site using processing scripts developed by the Netherlands site and ENIGMA [69].

**Table 4:** MRI Parameters

MRI Scanner	
Brazil	Philips Achieva 3.0 T
India	Philips Ingenia 3.0 T CX
Netherlands	GE 3.0 T Discovery MR750
South Africa	Siemens MAGNETOM Skyra 3.0 T
U.S.	GE 3.0 T SIGNA Premier
Head coil	32-channel or 48-channel^#^
MRI Sequences	
Structural T1	3D sagittal T1-weighted MP-RAGE according to ADNI-3 protocol (1 × 1 × 1 mm resolution)
rs-fMRI	T2*-weighted echo-planar images while subjects are awake and keep their eyes closed (10 min, TR = 2200 ms, TE = 28 ms, 3.3 × 3.3 mm in-plane resolution, 3 mm slices with 0.3 mm gap)
DWI	multi-shell single spin echo DWI (80 interleaved volumes: 7 b0, 25 b1000, 24 b2000, 24 b3000, 2.5 × 2.5 × 2.5 mm)
PSIR	T1-weighted image with improved SNR and gray-white matter contrast (1 × 1 × 1 mm resolution)

All sites use a 32-channel head coil, except NYC which uses a 48-channel head coil. *Abbreviations*: *rs-fMRI* Resting-state functional Magnetic Resonance Imaging, *DWI* Diffusion weighted Imaging, *PSIR* Phase sensitive inversion recovery

We chose standard imaging sequences for structural and functional imaging, which are commercially available or feasible to implement on most clinical scanners. Our rationale was that overly specialized paradigms that can be conducted only in a few WEIRD [27] laboratories will have limited clinical impact across most of the world. In contrast, if we identify robust biosignatures of psychopathology across countries/cultures using standardized imaging, our findings can then be leveraged by many.

#### Study flow

**Fig. 3:**
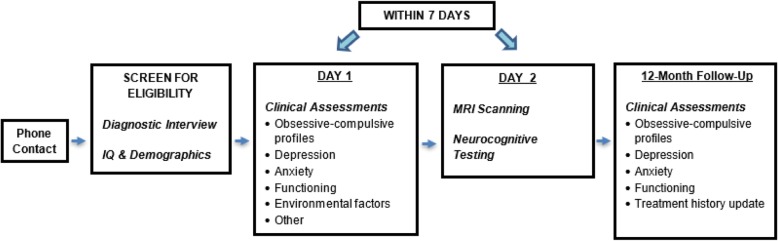
Study Design

The timing of the assessments and the flow of subjects through the study are outlined in Fig. 3; the specific measures used for each clinical domain are listed in Table 2. The clinical assessments, neurocognitive testing, and MRI scanning will be conducted within 7 days (and ideally within 48 h) to be able to correlate these measures.

### Study status

The study is ongoing and currently all sites are collecting data at the time of this paper’s submission.

### Data analytic plan and power analysis

We will collect data from a total of 600 individuals (250 OCD, 100 unaffected siblings, and 250 HC) across five sites. Our first aim is to identify reproducible neuroimaging signatures that distinguish individuals with OCD from unaffected siblings and healthy controls. To accomplish this aim, each imaging modality (anatomical, DWI, and rs-fMRI) will be analyzed using standardized protocols for uni-modal analyses; fusion of multi-modal imaging measures using modern machine learning or multilayer analyses [58, 133] will also be used to identify data-driven signatures that distinguish the groups. Our second aim is to link these neuroimaging signatures to behavioral performance on the cognitive tasks that probe these same circuits (Table 4) and to different clinical profiles commonly seen in individuals with OCD (e.g., different symptom dimensions, degree of insight, age of onset, comorbidity). We will also explore whether three environmental factors (childhood trauma, social economic status, and religiosity) moderate the link between our neuroimaging signatures and our OCD clinical and cognitive profiles.

With 250 medication-free OCD subjects, 250 demographically matched HCs, and 100 unaffected siblings, we will have 80% power while controlling for Type 1 error to 5% to detect effect size differences (i.e., group differences on a standardized scale) of Cohen’s d = 0.25 between OCD and HC groups, and d = 0.33 between unaffected siblings and either OCD or HC subjects. We note that even within sites (i.e., within each country) our sample size provides > 80% power to detect effect sizes of d = 0.57 between OCD and HC subjects and d = 0.80 when comparing either group to unaffected siblings. We will have 80% statistical power to test correlations of sizes as small as *r* = 0.12 across all subjects at all sites (*r* = 0.25 within site) and *r* = 0.18 across all OCD subjects at all sites (*r* = 0.40 within site). Previous studies, including our own, have found moderate to large effect sizes (i.e., d = 0.35–0.90) in neuroimaging measures corresponding to our OCD versus HC hypotheses, and recent studies have found strong correlations (e.g., 0.40 or greater) between neuroimaging measures and cognitive and clinical profiles [43, 47, 59]. Hence, the current study is powered to identify previously found or smaller effect sizes across all sites. Moreover, we expect statistical power for detecting OCD neuroimaging profiles to be increased through our fusion of multi-modal imaging data with machine learning statistical methods, which optimally combine all information on the same individual.

### Quality control

#### Overview

Each site will be responsible for all research procedures at its setting and will contribute to the cross-site workgroups as described below. The U.S. site is responsible to NIMH for the overall conduct of the study and will be directing the data management and statistical plan. The Netherlands site will oversee all imaging procedures, including harmonization of the imaging sequences, review of image quality, and image processing.

To ensure quality control and cross-site communication at all levels of the teams, we created clinical, neurocognitive, and imaging workgroup workgroups, each of which is led by one member of the executive committee and includes at least one member from each site. Speaking once or twice per month, each workgroup is responsible for quality control in its domain. Specific quality control procedures are described below.

#### Clinical assessments

Prior to enrolling study subjects, reliability of the clinical raters across all sites for our diagnostic and clinical measures was confirmed, following best practices used in prior NIMH-funded clinical trials. First, we created a manual to standardize administration of core clinical measures across all sites. The manual contains general instructions for administration as well as guidelines for rating specific items on each measure. Second, each site provided typed transcripts of interviews completed with actual patients, and all raters scored them to ensure that interrater reliability was high across sites. All raters were required to be reliable on three versions of each core clinical measure prior to study start. Third, to ensure ongoing interrater reliability throughout the recruitment period, clinical raters will re-rate a random sampling of transcribed interviews from all sites each year and attend monthly conference calls to discuss and resolve any discrepant ratings. A second call each month will be used to discuss any other clinical issues that arise, including questions related to recruitment and eligibility.

#### Neurocognitive testing

We created a manual of standard operating procedures for the computerized neurocognitive protocol used in the study. Sites bought laptops with the same specifications (e.g., resolution, screen size, processor, random access memory (RAM), operating system) and standardized the set-up of the testing rooms to ensure that tasks would be presented consistently across sites. Each site tested the protocol with at least five volunteers to ensure proper use and output of the protocol, and a member of the team also reviewed screenshots and videos of each task at each site to confirm standard presentation of stimuli. Throughout study recruitment, output from each neurocognitive task will be reviewed periodically (e.g., after the first 10 subjects at each site are enrolled, then after the next 20 subjects) to ensure ongoing data quality. Members of the neurocognitive workgroup attend up to two conference calls each month to review administration of the tasks, discuss quality control, and ensure that data are being properly recorded and stored.

#### Neuroimaging data

At study start, we harmonized MRI data collection across the five sites so that raw MRI data (anatomical, DWI and rs-fMRI) can be optimally pooled. We followed established methods used in other multi-center MRI studies to reduce between-scanner effects [34, 134–136]. These methods included: harmonization of scan sequence, brain coverage, and spatial resolution, as well as assessment of scan quality using two physical phantoms (i.e. National Institute of Standards and Technology (NIST) and Functional Biomedical Informatics Research Network (*fBIRN*) Agar). Two traveling humans were also scanned at all sites to assess within-subject scanner variability.

For continued quality assurance, physical phantoms will be scanned bi-monthly (NIST for geometry) and bi-weekly (fBIRN Agar for temporal stability). In addition, during the recruitment phase, all sites will send neuroimaging data of enrolled subjects to the Netherlands site within 48 h after a scan is acquired. The Netherlands site subsequently preprocesses incoming scans to check for correct spatial and timing parameters and to further ensure imaging quality during the recruitment phase by visual inspection and automated image quality measures of scanner or motion-related artefacts, scan coverage, and signal drop-out. Preprocessing and quality assurance is performed using open-source MRI processing suites such as FMRIB Software Library (FSL version 6.0.1, FMRIB, Oxford, UK), QUAD [137], and MRIQC [138]. Any anomalies are reported back to the site for follow-up.

## Discussion

Using harmonized methods for data collection and analysis, we will conduct the largest multimodal imaging and neurocognitive study in medication-free adults with OCD to date. Our study is designed to address a key question in the field: can we identify reproducible biosignatures of psychopathology that will change how we conceptualize mental illness, develop a mechanistic understanding of how current treatments work, and provide robust new targets for treatment development? As reviewed above, OCD provides an excellent test of this question, and the study described above represents the first step. Specifically, we will leverage our large diverse sample, multimodal imaging, and modern data-driven imaging methods [58, 133] to test whether we can empirically derive imaging signatures that reliably differentiate OCD patients from unaffected siblings and healthy controls.

With the increasing recognition of the global burden of mental illness [67, 139] and the fact that the International Classification of Diseases (ICD) will make OCD the lead disorder in a new cluster that includes OCD and related disorders [62], this is an opportune moment to focus the global health community on OCD by conducting an international study like this. Although there have been prior imaging and neurocognitive studies on OCD, ours will be the first to examine a very large sample of individuals who are free of medication and to use harmonized imaging methods and neurocognitive tasks in accordance with the RDoC domains. Our work will clarify whether OCD subjects across the globe show altered structure and function within frontal-striatal, frontal-limbic, and frontal-parietal circuits, as we hypothesize. If we see the structural abnormalities found in several meta- and mega-analyses [34, 35, 140, 141], including that of ENIGMA-OCD [36, 37], it will provide strong evidence for the link between these structural abnormalities and OCD psychopathology. Moreover, we will test how multimodal imaging measures are linked to discrete domains of neurocognitive or clinical profiles, enabling us to make new discoveries about the association between abnormalities in structural and functional connectivity and cognitive and clinical dimensions of OCD.

Including unaffected siblings in our sample will contribute important new information about the brain circuit alterations associated with OCD risk and resilience, and we plan to recruit a sufficient sample of unaffected siblings to have the statistical power to examine these questions in a definitive way. Moreover, we will explore the relationship between environmental factors (childhood trauma, socioeconomic status, religiosity) and the brain signatures of OCD, as well as their potential moderating effect on the relationship between these signatures and OCD neurocognitive and clinical profiles. Consequently, this study will advance our understanding of the socio-contextual factors that influence OCD risk, severity, and associated morbidity, providing additional mechanisms to target for treatment and prevention.

Finally, our study is designed to strengthen the public health impact of research through large-scale global collaboration. By linking our five research sites, we will be able to recruit a large and diverse sample to examine questions that no single site alone could address. Moreover, by leveraging our intellectual resources and methodological expertise, we seek to accelerate discovery toward a circuit-based approach to cognitive and clinical dimensions of OCD. In the process, we integrate two different perspectives: that of global mental health, which has been focused on building capacity and service delivery in low-resource settings and closing the research and treatment gap, and that of translational neuroscience, which is focused on discovery of fundamental brain processes and mechanisms underlying psychopathology and using this knowledge to transform symptom-based approaches to diagnosis and treatment [142]). We integrate these perspectives by selecting imaging and neurocognitive measures that can be used in diverse settings (the “World Health Organization [WHO] approach”) [66]; this way, our findings can be leveraged by many.

Empirically derived neural circuit taxonomies represent a new direction of discovery in psychiatry, and similar statistical methods have identified distinct biotypes in psychosis [143] as well as in depression and anxiety [144–147]. If we successfully link different brain circuit abnormalities to discrete cognitive and clinical profiles that are characteristic of OCD, we will be positioned for future study of how these brain circuit abnormalities develop during the course of disease, determine how they cut across traditional diagnostic boundaries, and use them as robust new treatment targets. Ultimately, this research could lead to objective methods for early diagnosis and intervention as well as to transdiagnostic treatments, with discoveries that will be relevant to populations across the globe.

## Data Availability

Not applicable.

## Abbreviations

BABS: Brown Assessment of Beliefs Scale
CBT: Cognitive-behavioral therapy
CFI: Cultural Formulation Interview
CSTC: Cortico-striatal-thalamo-cortical
DWI: Diffusion weighted imaging
DY-BOCS: Dimensional Yale-Brown Obsessive-Compulsive Scale
ENIGMA: Enhancing Neuroimaging and Genetics through Meta-analyses
*fBIRN*: Functional Biomedical Informatics Research Network
HAM-A: Hamilton Anxiety Rating Scale
HAM-D: Hamilton Depression Rating Scale
HCs: Healthy control subjects
IQ: Intelligence quotient
MDD: Major depressive disorder
MRI: Magnetic resonance imaging
NIST: National Institute of Standards and Technology
OBIC: OCD Brain Imaging Consortium
PIs: Principal investigators
PSIR: Phase sensitive inversion recovery
RAM: Random access memory
RDoC: Research Domains Criteria
rs-fMRI: Resting state functional MRI
SCID: Structured Clinical Interview for DSM-5
SES: Socioeconomic status
USP-SPS: University of São Paulo Sensory Phenomena Scale
WAIS-IV: Wechsler Adult Intelligence Scale Fourth Edition
WAMI: Work and Meaning Inventory
WASI-I: Wechsler Abbreviated Scale of Intelligence First Edition
WASI-II: Wechsler Abbreviated Scale of Intelligence Second Edition
WEIRD: Western, educated, industrialized, rich, and democratic
WHODAS: World Health Organization Disability Assessment Schedule 2.0
YBOCS: Yale-Brown Obsessive-Compulsive Severity Scale
